# Implementation of Evidence-Based Psychological Treatments to Address Depressive Disorders: A Systematic Review

**DOI:** 10.3390/jcm14176347

**Published:** 2025-09-08

**Authors:** Rosa Lorente-Català, Amanda Díaz-García, Irene Jaén, Margalida Gili, Fermín Mayoral, Javier García-Campayo, Yolanda López-Del-Hoyo, Adoración Castro, María M. Hurtado, Caroline H. M. Planting, Azucena García-Palacios

**Affiliations:** 1 Department of Clinical and Basic Psychology and Biopsychology, Faculty of Health Sciences, University Jaume I, 12071 Castellon, Spain; rlorente@uji.es; 2 Department of Psychology and Sociology, Universidad de Zaragoza (Campus Teruel), 50009 Teruel, Spain; amandadiaz@unizar.es; 3 Department of Developmental, Educational, Social and Methodology Psychology, Faculty of Health Sciences, University Jaume I, 12071 Castellon, Spain; ijaen@uji.es; 4 Research Group of Mental Disorders of High Prevalence (TRAMAP), Research Institute of Health Sciences (IUNICS), University of Balearic Islands, 07122 Palma de Mallorca, Spain; mgili@uib.es (M.G.); a.castro@uib.es (A.C.); 5 Health Research Institute of the Balearic Islands (IdISBa), 07120 Palma de Mallorca, Spain; 6 Primary Care Prevention and Health Promotion Research Network, Red de Investigación en Actividades Preventivas y Promoción de la Salud (RedIAPP), 28029 Madrid, Spain; 7 Mental Health Department, Institute of Biomedicine of Malaga, University Regional Hospital of Malaga, 29010 Malaga, Spain; fermin.mayoral.sspa@juntadeandalucia.es (F.M.); marienahurtado@gmail.com (M.M.H.); 8 Instituto de Investigación Sanitaria de Aragón (IIS Aragón), 50009 Zaragoza, Spain; jgarcamp@gmail.com (J.G.-C.); ylopez.iacs@gmail.com (Y.L.-D.-H.); 9 Department of Psychology and Sociology, University of Zaragoza, 50009 Zaragoza, Spain; 10 GGZ inGeest Specialized Mental Health Care, 1070 BB Amsterdam, The Netherlands; c.planting@ggzingeest.nl; 11 CIBER Physiopathology Obesity and Nutrition (CIBERobn), Carlos III Health Institute, 28029 Madrid, Spain

**Keywords:** evidenced-based psychological treatment, implementation, depression, psychotherapy

## Abstract

**Background:** The depressed population needs to be treated and they do not have access to evidenced-based psychological practices (EBPPs). The consequences lead to significant daily impairments and huge economical costs. A large amount of research has focused on the demand for a more extensive use of EBPPs. However, despite these practices being essential to the mental health system, EBPPs are poorly applied in clinical settings. This situation has led to the development of Implementation Research (IR), a scientific field that aims to address the challenge of translation and identify the factors involved in the implementation process. Several implementation studies have been carried out in the field of health. However, the evidence from implementation studies of psychological treatments addressing depression has not yet been summarized. The aim of this study is to conduct a systematic review to assess implementation studies that use EBPPs to address depression. **Methods:** A systematic review was conducted following the PRISMA guidelines, including implementation studies that applied EBPPs to address depressive disorders. The following databases were used: PubMed, Embase, APA PsycInfo, Cochrane Central, Scopus, and Web of Science. Two independent reviewers revised the studies to determine whether the eligibility criteria were met. **Results:** A total of 8797 studies were identified through database searches. After removing duplicates, a total of 3757 studies were screened based on titles and abstracts. Finally, 127 full-text articles were reviewed, yielding 31 studies that satisfied the inclusion criteria. **Conclusions:** This review offers valuable insights into the current state of IR in the implementation of EBPPs for treating depressive disorders. It underlines the necessity for a standardized nomenclature for study designs within the realm of IR and emphasizes the potential of hybrid efficacy–implementation studies to help close the gap between research and clinical practice. Despite the challenges encountered, this review points to a positive outlook for the use of IR in clinical psychology. A gradual adoption of IR is likely to strengthen its role in psychology and support the development of more effective strategies for implementing evidence-based interventions in clinical settings.

## 1. Introduction

The urgency to address depression is growing with each passing day. Empirical data supports the forecast that by 2030, depression will become the leading contributor to the global burden [1]. Despite the persistent alerts and the calls for intervention, the prevalence of depression continues to remain high and shows an increasing trend [2]. Depression is assumed to significantly impair daily life, well-being, and social functioning. It also leads to high annual costs, yet access to treatment is limited [3]. In fact, even though there are effective treatments available for depression [4], only around a quarter of the depressed population in high-income countries and a mere 3% in low- and lower middle-income countries [5] receive treatment that is minimally adequate.

This problematic situation could be partially explained by the know–do gap. This gap refers to the disparity between what is known through research and what is actually put into practice in real-world settings [6,7,8]. Indeed, there is a time-lapse of 17 to 20 years for the implementation of Evidence-Based Psychological Practices (EBPPs) [9]. Although these practices offer crucial advantages for the population, this time-lapse prevents their implementation, and the quality of the treatments does not grow with the scientific knowledge. Furthermore, it has been estimated that less than 10% of psychologists use EBPP manuals when EBPP reaches clinical settings [10].

The science of Implementation Research (IR) aims to tackle the challenge of translation [11] by studying the strategies and factors influencing the process of translating EBPPs into daily practices [12]. Focusing on the need to promote and ensure the application of scientific innovations, the main goal of IR is to improve the quality and effectiveness of interventions, identifying the factors involved in the implementation process. IR aims to transfer science from the laboratory to health care services, maintaining its validity and providing specific strategies to carry out this process [13,14].

Despite a growing body of literature on the therapeutic changes experienced by the population with different mental disorders [15], almost no research attempts to understand the dynamic interactions between individuals and the context in which they receive treatment, and how these interactions may influence individual improvement [9,16]. Unlike laboratory simulations conducted far from clinical settings, implementation studies are context-specific. These studies focus on determining the reasons for the effectiveness of a treatment in a specific context as well as the factors that influence it. Therefore, the main objective of implementation studies is to determine the barriers and facilitators in each context and develop specific implementation strategies for a specific treatment. The implementation process is developed by searching for the applicability of psychological interventions in care settings [12,17].

Due to IR proliferation, the need for a specific theoretical framework for developing implementation studies has been identified [18,19,20]. Some of the theoretical frameworks in the health field are (1) RE-AIM (Reach, Effectiveness, Adoption, Implementation, and Maintenance), which focuses on the fidelity of the intervention in these five dimensions [21]; (2) PARISH (Promoting Action on Research Implementation in Health Services), which evaluates the quality of the study related to the scientific evidence supporting the efficacy, the context, and the ease of carrying out the implementation [22]; (3) EPIS (Exploration, Preparation, Implementation, and Sustainment), which assesses the implementation process in four phases: the treatment selection process, the application plan, its implementation, and the maintenance of the intervention over time [23]. Despite the need for theoretical frameworks to promote the generalization and comparison of the results, their diversity hinders IR cohesion. A significant overlap is observed between more than 60 theoretical implementation frameworks and more than 70 implementation strategies, yielding a variety of terminology addressing similar constructs [20,24,25,26]. To overcome the over-production of theoretical frameworks, Damschroder and colleagues [17] developed a new integrative framework based on the review by Greenhalgh and colleagues [27], called the Consolidated Framework for Implementation Research (CFIR). The CFIR is a meta-theoretical framework that gathers 39 constructs in five domains (intervention characteristics, the inner and outer context, the individuals involved, and the implementation process). The primary objective of the framework is to assess the constructs that exert influence on the implementation process, discerning what works in what context and why it works. The constructs work as mediators and moderators that influence the implementation’s effectiveness at different levels of a treatment in a specific context [17]. Consequently, the overall structure of CFIR serves as a valuable tool for investigating critical issues that are inherent to any study or evaluation of implementation being widely applied in guiding the implementation process across a diverse range of research studies and environmental contexts [28].

To address the psychological needs of the population, it is essential to account for the application of evidence-based interventions for each psychological problem and consider the contextual characteristics as essential factors that determine the application of the intervention. Nevertheless, contextual factors or EBPPs are poorly applied in clinical settings, with scientific advances not being easily transferred to daily practice [29,30]. IR offers the methodology and scientific approaches to address this problem. Psychological researchers are increasingly recognizing the significance of advancing implementation studies, as emphasized in a wide range of published works. However, it remains evident that a substantial proportion of researchers have yet to engage in such endeavors [9,31]. The controversy and misinterpretations highlight the importance of establishing an accurate IR modality in clinical psychology. In the field of health, previous systematic reviews have been carried out on treatment implementation studies [20,32,33,34,35,36]. However, none of the included studies specifically approached the area of mental health. In terms of general mental health, two systematic reviews have been carried out [32,33]. In the field of children and adolescents, we found a review of dissemination and implementation studies within the area of mental health. This systematic review, made up of 44 studies, only included 10 treatment studies, and the rest were prevention studies. Furthermore, most of the studies applied dissemination strategies, and implementation was not addressed in detail by the selected studies [30,31]. In another review, researchers evaluated the effectiveness of strategies for the implementation of EBPPs. However, of the 11 selected studies, the majority considered dissemination procedures, raising the question of whether this review should be considered within IR [33]. Considering implementation focused on addressing depression, a recent scoping review was identified [36]. However, the scope of the review focused on e-mental health, encompassing exclusively the application of EBPPs through online platforms, which limited the review. Furthermore, within the cohort of 33 studies that were incorporated, only 12 studies undertook interventions addressing depression, with the remaining studies targeting anxiety-related conditions [36].

It is equally important to highlight that reviews are typically conducted in a generalized manner, with most studies not placing significant emphasis on the evaluation of the implementation process.

Based on the previously discussed information and the apparent lack of systematic reviews focused on implementation studies that address depression, we conducted a systematic review with the aim of assessing implementation studies that target depression.

Specifically, this systematic review aimed to identify implementation studies that were conducted to address depression, the EBPP that was implemented, and the theoretical frameworks employed in these implementation studies.

IR is a broad field typically categorized into two types of studies: those focused on implementing specific EBPPs within particular contexts and those geared towards evaluating the barriers and facilitators, as well as strategies, aimed at enhancing the implementation process [37]. This review focuses on studies that have reported the actual implementation process and have actively examined the uptake of interventions, rather than delving into the barriers within a specific context. Consequently, unlike previous reviews, which offered only general information, we provide a comprehensive assessment of the included studies and report the implementation quality of the studies based on the CFIR framework.

To the best of our knowledge, no other reviews assessing the quality of implementation studies that address depression have been published. This dearth of literature on existing reviews could potentially hinder our understanding of the current state of IR in the field of clinical psychology. Consequently, it remains unclear to what extent and in what manner EBPPs are being implemented within healthcare settings.

## 2. Methods

This systematic review was conducted in accordance with the guideline of the Preferred Reporting Items for Systematic Reviews and Meta-Analyses (PRISMA) (see Supplemental Material for PRISMA checklist) [38,39].

The systematic review protocol was registered in the International Prospective Register of Systematic Reviews (PROSPERO: CRD42022319866).

### 2.1. Searches

The search strategy was established following Cuijpers’ guide [40] for the development of systematic reviews and meta-analyses in mental health. The entire search process was conducted following the guidelines and mentored by Information Specialists in Mental Health (CH-M). The first search was performed on 14 March 2022, and it was subsequently updated on 19 June 2024 to ensure the inclusion of the most recent studies.

We conducted a systematic search of the peer-reviewed literature on all the available implementation studies applying EBPPs to address depressive disorders. The following databases were used: PubMed, Embase, APA PsycInfo, Cochrane Central, Scopus, and Web of Science. Additional studies were also retrieved from Google Scholar and references from relevant articles. If full-text versions were not available or data were missing or unclear, we contacted the respective author.

The research question was defined through the PICO elements (Participants, Interventions, Comparisons, and Outcomes) [40]. The review focused on implementation studies of services or organizations (Participants) that implemented any psychological EBPP to address depressive disorders (Intervention), with no comparison established (e.g., intervention vs. no intervention, intervention vs. intervention) (Comparison), reporting outcomes related to the implementation process (Outcome). According to the established PICO analysis, the following components were established: (1) depressive disorder; (2) psychotherapy; and (3) health plan implementation. The complete search strategy applied in each database is found in the Supplementary Materials. Search terms were combined using the Boolean “OR” between synonyms and the Boolean “AND” for non-synonymous terms.

### 2.2. Study Inclusion and Exclusion Criteria

This systematic review identified English-language implementation studies of EBPPs that address depressive disorders. The following inclusion and exclusion criteria were established.

Inclusion criteria: (1) Implementation studies understood as the definition already established. (2) The main treatment implemented had to be a psychological intervention. (3) The psychological intervention had to be evidence-based. (4) The psychological intervention addressed a depressive disorder, specifically a major depressive disorder or persistent depressive disorder. (5) The psychological disorder had to be diagnosed using any recognized diagnostic criteria or screening tools to determine symptom severity.

Exclusion criteria: (1) Studies did not report implementation outcomes. (2) The main treatment implemented was pharmacological or focused primarily on physical health. (3) The study used unvalidated instruments to diagnose or did not screen for symptoms severity. (4) The psychological intervention was non-evidence-based. (5) The literature corresponded to study protocols, systematic and/or meta-analysis. (6) The full-text article was not available through Open Access or library loaning services. (7) The full-text article was not available in the English language.

In this review, implementation studies are defined by the uptake of an EBPP in a specific context [41]. In accordance, three essential criteria are needed to establish an implementation study: the implementation process, implementation site, and the EBPP, which allows us to differentiate the intervention-level activity and the implementation-level activity [12]. At the implementation level we find the implementation outcomes, in this case defined following Proctor’s Taxonomy [42]. The taxonomy is composed of eight implementation outcomes: acceptability, adoption, appropriateness, feasibility, fidelity, implementation cost, penetration, and sustainability. In this review, implementation studies were detected by following the key questions of Peters and colleagues to assess research designs on IR and check the three essential criteria (implementation process, site and EBPP) [43].

To develop the evidence-based criteria, the guidelines of the National Institute for Health and Care Excellence (NICE) together with the literature review of EBPP in the treatment of mental disorders were applied [44]. Furthermore, the cited evidence from the authors of the studies that supported its efficacy or effectiveness was considered.

Following Landsverk [45], our search allowed psychological interventions that occurred outside of traditional mental health service settings if the primary focus was on mental health treatment (e.g., schools).

No restrictions were applied regarding the participants’ age, the duration of the intervention, the delivery format (group or individual), the frequency of the sessions, the use of comparators (treatment as usual or waiting list), or the type of outcomes evaluated (e.g., implementation, service, or clinical), nor were there any restrictions on the year of publication of the study.

This systematic review was limited to English-language literature in peer-reviewed journals.

### 2.3. Data Extraction Strategy

Two independent reviewers (RL-C and AD-G) completed database searches, and the results obtained were entered into Hubmeta, a web-based data entry system for Meta-analysis and Systematic Reviews, where the following steps were conducted. All duplicates were removed. The same reviewers independently screened titles and abstracts to identify potentially relevant articles. All the selected articles in this first round were assessed by deeply reading the full text. Those that met the established criteria were selected as eligible studies. Any disagreements between the two reviewers (RL-C and AD-G) were resolved by a third senior researcher (AG-P). The study selection process and the justifications for exclusions at each level of the process were recorded in the PRISMA flowchart [38]. The following information was extracted from the studies: authors, year of publication, country, study design, implementation outcomes reported, patients, implementers, the theoretical framework in the field of IR, characteristics of the Evidence-Based Intervention, the setting, the aim and the units of analysis.

### 2.4. Study Quality Assessment

The quality of reporting on implementation outcomes was assessed in the 5 domains and 39 constructs of the CFIR theoretical framework [17]. CFIR was selected as the theoretical framework used to guide the evaluation process for different reasons. First, the integrative characteristics of CFIR address large numbers of different theoretical frameworks applied in the literature [17]. Second, the CFIR is not only categorized as a determinant implementation framework, but also as an evaluation framework [19], defined as a tool to code and rate qualitative data to enable comparisons across studies [17]. Consequently, the scientific value of these studies for IR was established through a qualitative assessment using the CFIR. Two reviewers (RL-C and IJ) conducted information data extraction and disagreements were resolved by a third senior researcher (AG-P).

## 3. Results

### 3.1. Review Statistics

**Figure 1:**
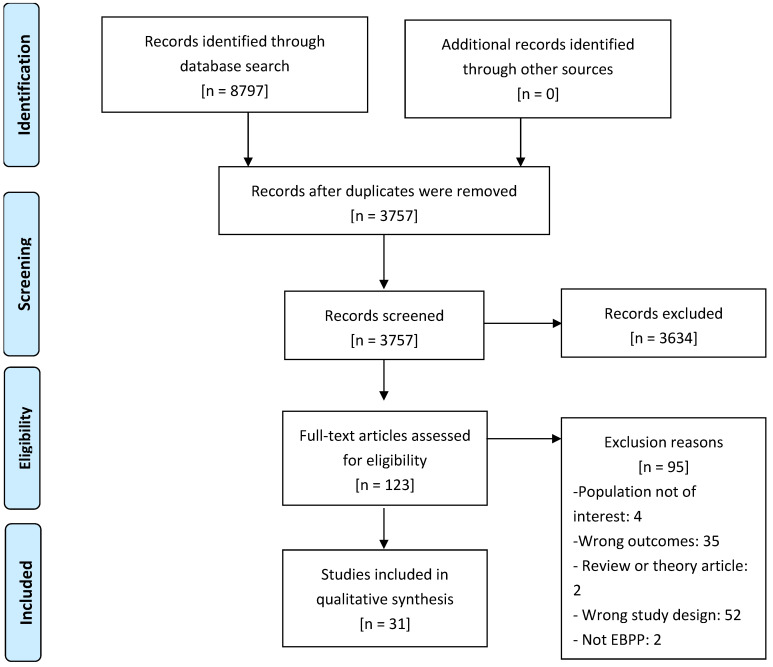
PRISMA diagram of study selection.

A total of 8797 studies were identified through database searches conducted on 14 March 2022 (PubMed = 1619; Embase = 1425; APA PsycInfo = 83; Cochrane Central = 1308; Scopus = 1758, and Web of Science = 1856). Of these 8797 studies, 757 were duplicate retrievals and removed, leaving 3757 unique articles reviewed based on titles and abstracts. The next step was the exclusion of articles using the title and abstract and the inclusion criteria analysis. Of these 3757 studies, 3634 were excluded. Subsequently, the review resulted in 127 potential articles for full review. Finally, 31 studies were selected for final inclusion in this systematic review (see Figure 1 for PRISMA Diagram).

### 3.2. Characteristics of Studies

Table S1 (Supplementary Materials) provides an overview of the main results obtained in the included studies.

#### 3.2.1. Research Objective and Units of Analysis

The main objectives of the implementation studies and their basic units of analysis (i.e., patients, therapists, caregivers) appear in Table 1.

As some of them present hybrid designs, in some cases, the main goal was still considered the effectiveness of the practice [51,53,58,61,63,64,69,72,73].

Nevertheless, all the studies focused on assessing the different factors influencing the implementation process. The emphasis on IR varied among studies, with the majority having multiple focuses.

Most of them considered feasibility [46,49,56,58,59,60,61,64,69,70,72,73] and acceptability [46,51,53,55,59,60,63,65,69,72] to be the main goal. Other studies focused on the perspectives of the implementation process or the EBPPs at all levels, including implementers and patients [47,49,50,63,65,67].

Some studies did not only focus on EBPP implementation but also assessed the uptake of implementation strategies for a practice [54,63,66,70].

Other studies focused on detecting the barriers and facilitators to implement a practice in a specific context [1,50,56,71] or explained the implementation process in real-world settings [48,68], establishing the guidelines for future studies. In the implementation process, the implementation training was considered by different studies [52,58,63,66,74,75,76], such as the fidelity when implementing the EBPPs [53,60,62]. Not only was the fidelity of the intervention taken into consideration, but also the extent of adoption, penetration, and sustainability were the focal points of certain studies [62,63,65].

In terms of the units of analysis, an array of dimensions were evaluated to rate the implementation process. The predominant focus of the studies was on patients [46,48,49,50,51,53,54,55,56,58,59,60,61,62,64,65,68,69,70,72,73,75], encompassing assessments related to implementation and effectiveness outcomes. Another crucial unit of analysis was related to implementers or stakeholders, with many studies distinguishing between various roles such as social workers, therapists, nurses, and peer-counselors [46,47,48,49,50,52,53,54,56,57,58,59,60,61,62,63,64,65,66,67,69,70,71,74,75,76]. An additional dimension involved caregivers [48,53,60,64,65] as a distinct unit of analysis. Lastly, when considering the contextual factors, organizational aspects were also subject to evaluation [52,54,56,58,62,65,71,74].

#### 3.2.2. Design

In the current systematic review, various study designs were employed. Three studies were structured around the Type I hybrid design, emphasizing effectiveness, with implementation as a secondary goal [55,59,61]. Five studies adopted a Type II hybrid design, emphasizing both effectiveness and implementation [48,49,53,60,66], while one study utilized a Type III hybrid design, with the assessment of the implementation being the main goal and effectiveness a secondary goal [50]. Other designs encompassed randomized trials [56,58,62,69,71,72,76] and non-randomized studies [46,54,73], along with an uncontrolled case study [65].

Additionally, three studies adopted longitudinal designs [51,63,70] and one study employed a large-scale, multi-site, multi-cohort approach [75]. The review also included studies focused on process evaluation [57] and pure implementation studies [68,74].

#### 3.2.3. Implementation Outcomes

Implementation outcomes were established following Proctor’s taxonomy. Table S1 (Supplementary Materials) presents the implementation outcomes addressed by each study. It is worth noting that studies typically assessed more than one outcome. Below we provide details on the number of studies that examined each implementation outcome.

Nineteen studies assessed acceptability, defined as the perception that a practice is agreeable or satisfactory [46,47,50,51,52,53,55,57,58,59,60,62,63,65,66,67,68,69,72,73]. Adoption, defined as the initial decision or the intention to uptake the practice or innovation within the setting, was assessed by eight studies [54,57,62,63,65,66,73,74]. Appropriateness, which refers to the perceived fit, relevance or compatibility of the practice in a specific setting, provider or consumer, was considered by five studies [47,52,57,65,70].

In the majority of studies, feasibility, which assesses the degree of successful practice implementation, was evaluated, with a total of twenty-six studies addressing this aspect [46,47,48,49,52,53,54,55,56,57,58,59,60,61,63,64,65,66,68,69,70,71,72,73,74,75]. Seventeen studies considered fidelity, which assesses the degree to which a practice is implemented according to the established protocol [46,48,49,50,52,53,54,56,58,60,62,66,69,71,73,76]. Implementation cost, examining the cost impact of the implementation process, was considered in four studies [54,57,64,65]. Penetration, defined as the integration of a practice within the system of a specific setting, was assessed by five studies [54,55,56,63,65]. Lastly, nine studies assessed sustainability, which measures the extent to which a new practice is maintained over time in a specific setting [54,55,57,62,63,65,71,73,74].

#### 3.2.4. Characteristics of the Population

Regarding age, two studies addressed depression in children [65,71], an important number of studies were conducted with young people, including adolescents [48,58,60,62,64,69], students [53,72] and university students [51,76], and one study specifically targeted the older population [50].

Concerning the specificity of the samples, one study was conducted with an incarcerated population [59], one with company employees [56] and four studies were with Veterans (VA) [49,54,61,75].

Studies that targeted depression with the presence of another special condition included HIV/AIDS [46], pregnancy, post-partum and perinatal depression [47,52] and patients with heart failure and/or chronic obstructive pulmonary disease [67]. Furthermore, some studies addressed depression in comorbidity with other psychological conditions such as behavior disorders [48], substance abuse [63] and anxiety or PTSD [51,55,74].

#### 3.2.5. Theoretical Framework

While some studies explicitly reference and base their research on guiding theories or theoretical frameworks related to IR, others either do not adhere to any theoretical framework or do not specify the one they are using [46,47,48,50,51,53,56,58,60,61,62,64,68,69,70,74,75,76].

The taxonomy of implementation outcomes by Proctor [42] was applied in three studies [55,63,65]. Other well-established frameworks were applied, such as RE-AIM [21], which focuses on the steps for an implementation process with the fidelity of a practice; it was used in three studies [54,67,73]. Two studies considered the CFIR, which explores the different constructs that intervene in the implementation process [57,59]. Another two studies were guided by the “Find, Organize, Clarify, Understand, Select—Plan, Do, Study, Act” cycle (FOCUS-PDSA) [77], which establishes the process to identify problems and select, test and use a solution to promote rapid quality improvement [49,54]. Other studies used the Active Implementation Framework [52] and the Normalization Process Theory [78], which study the processes behind the final integration of a practice [63] and the Exploration, Preparation/Adoption, Implementation and Sustainment (EPIS) [79], which addresses the factors to consider during the implementation process [71]. Another study [66] was guided by PARISH, through which three aspects are considered: the EBT, the contextual characteristic and the implementation facilitation process [80].

#### 3.2.6. Treatment Characteristics

All the interventions employed in the studies adhered to the fundamental tenets of IR and were categorized as EBPPs. Among these interventions, Cognitive-Behavioral Therapy (CBT) was the most widely implemented [61,62,66,74], sometimes being group-based [53], internet-based, such as the intervention Beating the Blues [57,72], or in a brief format [66,67]. Additionally, some interventions either utilized CBT principles or incorporated components of CBT, exemplified by the Healthy Emotions Program [48], Mamma Mia (an online program) [52], or Creating Opportunities for Personal Empowerment (COPE) [64]. Others used CBT components in combination with mindfulness strategies such as Mindfulness and Acceptance and Commitment Therapy for Depression (ACT-D) [75], Acceptance-based group therapy [55] or MindBalance [68].

Interpersonal Therapy (IPT) was featured in several studies [46,47,59,76] along with IPT-Adolescents Skills Training (IPT-AST) [60]. Other studies also included interventions related to bonding aspects, specifically with the family, with Based Family Therapy [58] and Multi-Family Psychoeducational Psychotherapy (MF-PEP) [65].

One study implemented Happy@Work, an online intervention based on Problem Solving strategies and Cognitive Therapy (CT) [56], as well as Collaborative Care Management (CCM) [54].

Inter-personal Counseling (PC) and Brief Psychosocial Support (BPS) [69] were also utilized.

Three studies focused on Behavioral Activation (BA) [63] techniques using interventions such as System Activation Method (SAM) [50] or Step-by-Step based on BA [73].

We found three different transdiagnostic interventions: UniWellbeing, which is online-based [51], Common Elements Treatment Approach (CETA) and EMOTION [70,71].

Finally, one study included a psychodynamic perspective with the Brief Dynamic Interpersonal Therapy (BDIT) [49].

#### 3.2.7. Study Settings

Regarding the countries where the studies were carried out, the majority of them (16 studies) were conducted in the United States (US) [48,49,53,54,55,59,61,62,64,65,66,67,70] followed by three studies in Norway [52,58,71] and two in the Netherlands [50,56], Finland [63,69] and United Kingdom (UK) [60,68]. Other included countries are South Africa [46], Israel [47], Australia [51], Canada [57] and China [73].

At the contextual level, most of the studies were performed in healthcare settings, such as clinics, mental health centers, primary care settings, psychiatric and ambulatory units, etc. [17,47,48,50,51,52,54,55,60,62,63,64,65,67,68,70,71,72,73,76], including specific VA clinics [49,75].

Six studies were conducted in educational settings, including schools, universities, and college counselling centers [51,69,71,72,73,76]. One study was conducted in international companies [56] and another study in state prison systems [59].

### 3.3. Qualitative Assessment

The implementation quality of the studies was assessed using the CFIR framework. Table S2 (Supplementary Materials) reports the constructs considered in the implementation process for each study.

The first domain, characteristics of the intervention, comprised eight constructs. One study assessed all the constructs, nine studies assessed half or more of them, twenty studies considered one to three constructs and one study considered none of the constructs. The most valued constructs were trialability and adaptability, addressed in eighteen studies. Nevertheless, only one study considered the intervention source.

In the case of the outer context, six studies assessed half of the four constructs in this domain. Ten studies at least assessed one construct and fifteen did not consider any of them. The construct of patients’ needs and resources was the most assessed in this domain (15 studies). None of the studies considered peer pressure.

The third domain, the inner context, is composed of 14 constructs. One study considered all the constructs and six studies assessed half or more than half of them. Three studies considered six, three considered five, and the rest considered one to four constructs. Only one study considered none of the constructs. The most considered were access to knowledge and information (28 studies) and available resources (26 studies), whereas leadership engagement was only considered in two studies.

The characteristics of individuals, the fourth domain, addresses the characteristics of the people involved in the implementation process through five constructs. One study examined all the constructs, and out of those, eight studies focused on a maximum of three constructs. Thirteen studies examined one to two constructs, while nine studies did not consider any. Knowledge and beliefs about the intervention were the most considered. By contrast, only two studies considered self-efficacy and three considered the identification with the organization.

The fifth domain encompasses the implementation process across eight constructs. Out of these, eighteen studies accounted for half or more of the constructs, seven studies considered two or three, and three studies focused on only one. Notably, three studies did not consider any of the constructs. Reflecting and evaluating emerged as the most extensively evaluated aspect, specifically by twenty-two studies. In comparison, opinion leaders were not considered in any study.

**Figure 2:**
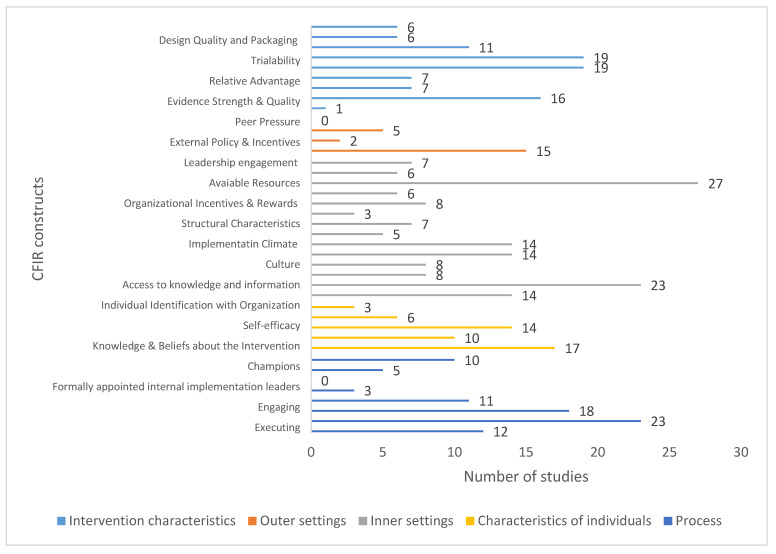
Studies that assessed each construct in the five domains of the CFIR.

To obtain a general impression of the constructs addressed in the studies, they have been assessed globally and represented in Figure 2.

## 4. Discussion

EBPPs have shown efficacy in addressing depressive disorders in several controlled trials [81]. However, access remains limited, highlighting the need for effective implementation strategies. IR provides the procedures to adapt EBPPs in different contexts. This review assessed the current state of IR in implementing psychological treatments for depression.

The search identified a total of thirty-one studies that focused on the implementation process of addressing depression through EBPP. Compared to medicine, research in psychology is still scarce [20,34], but the growing number of identified protocols indicates increasing interest in this field, even if they were excluded here due to criteria.

Although IR literature is expanding, conceptual and terminological inconsistencies persist. Many studies equated implementation with application, overlooking its broader meaning as the integration of interventions within specific contexts [12,14]. This reflects a lack of awareness of IR as an emerging science [8,27,82].

In a similar way and regarding the IR theoretical framework, the diversity in these frameworks and the theoretical divisions are evident [20,24,25,26]. Indeed, twenty of the included studies did not specify or consider any framework. Therefore, it is challenging to compare and evaluate the results, which makes it difficult to assess the state of this science in the field of clinical psychology. This underlines the urgent need for a common, integrative framework [19,20,83].

Hybrid designs were also poorly specified, often due to lack of guidance and inconsistent terminology [41,84]. In this review, the hybrid type was established post hoc using Curran’s categorization [41].

Regarding the contextualization, a variety of countries conducted studies. Nevertheless, there is a clear predominance of the US. The high concentration of implementation studies in this country is not accidental. It may be due to the inequalities in access to health and the high prevalence of specific pathologies such as PTSD and emotional disorders in specific settings like VA [85].

In relation to the specific context, IR has the objective of bringing the most appropriate treatments to the groups that need them most [11,12,13,14]. The studies included in this systematic review are consistent with this statement, given that the implementation process occurs in contexts with the greatest difficulties in accessing mental health services (e.g., lower secondary schools, prison systems, companies, etc.).

An essential aspect in implementing these interventions is the consideration of patient and professional characteristics, as well as contextual factors when selecting EBPPs. This highlights the significance of IR guidance. IR emphasizes the importance of the therapist’s identification with and attitude toward the intervention in the implementation process, as the implementation may not proceed effectively if it does not align with the therapist’s approach and methodology [86].

When implementing EBPPs, various implementation outcomes were considered [42]. We observed that most studies primarily concentrated on outcomes related to the early phases of the implementation process, such as acceptability and feasibility. These results emphasize the need to conduct comprehensive studies that cover all implementation outcomes, with a specific focus on achieving the long-term sustainability of interventions within specific settings [87]. These findings are consistent with previous reviews, which also highlighted a predominant focus on the dissemination phases of implementation while largely remaining at preliminary stages [30,31,32,33,36].

While broader studies within IR are needed, we have observed that this field facilitates the incorporation of a diverse array of implementation settings, outcomes, and, notably, a multitude of distinct objectives. These research objectives extend beyond mere effectiveness results or implementation outcomes. Indeed, they go deeper into aspects such as the implementation of facilitation strategies, the perspectives of various stakeholders, the identification of barriers and facilitators or the consideration of different units of analysis. Therefore, IR leads to a more comprehensive understanding of the intricacies of how the population can access EBPPs.

Regarding implementation quality, we found inconsistencies in the use of CIFR constructs. Intervention characteristics such as complexity, quality of presentation, and costs were rarely evaluated, even though these directly affect adherence and feasibility [17]. By contrast, treatment efficacy evidence was strong, positively influencing implementation but insufficient to guarantee adherence.

A significant number of studies assessed patients’ needs and resources, aspects considered essential for effective implementation, as they address both patient satisfaction and tools for therapists. However, as we stated earlier, the context is established as a key differential aspect between efficacy and efficacious studies and implementational studies [12] and overall, the assessed studies present significant deficiencies in the evaluation of the context, both internal and external.

Finally, a clear lack in the implementation process was observed in the underestimation of the characteristics of the people who carry out the intervention. The tip of the iceberg of the intervention consists of therapists’ actions. Not considering therapists as an implementation factor means ignoring the essential vehicle for transferring EBPPs to the clinical setting. However, this deficiency is partially mitigated in some studies through the evaluation of the implementation execution and the continuous feedback in the domains of the process. The exploration of the level of fidelity of the intervention through different constructs makes the studies gain quality in terms of their implementation. In fact, it has been observed that intervention training is not enough to ensure its proper implementation, and the evaluation of fidelity and promotion through follow-ups are necessary to ensure the effective implementation of treatments [88].

### 4.1. Strengths and Limitations

To the best of our knowledge, this is the first systematic review of IR in the implementation of psychological interventions to address depressive disorders. Furthermore, it assesses the quality of studies based on a well-established theoretical framework (CFIR). However, the assessments made in the present study must be considered in light of certain limitations. (1) The deficiencies of the studies that did not meet the established criteria should be evaluated to obtain a more comprehensive approach to IR. Assessing the limitations of these studies may highlight issues to consider in the implementation of interventions. (2) The review was limited to psychological treatments, which may have influenced the assessment of IR in mental health. It would be necessary to consider other types of approaches, such as studies carried out with preventive interventions. (3) Although the literature review was exhaustive and meticulous, some studies may not have been found and, consequently, were not included in the systematic review. (4) The review was restricted to English-language studies, which may have excluded relevant research published in other languages. (5) The diverse and inconsistent utilization of theoretical frameworks and terminology associated with implementation outcomes posed a challenge when categorizing studies according to Proctor’s taxonomy and the CFIR framework. Moreover, in certain cases, CFIR constructs may have been implicitly addressed in studies without being explicitly mentioned, potentially leading to misclassification. It should be considered that any instances of unclear classification were thoroughly deliberated by a panel of three authors. (6) The absence of a meta-analysis limits the ability to quantitatively synthesize results across studies, potentially affecting the generalizability of findings.

### 4.2. Future Directions

This systematic review raises an essential question: Is scientific evidence generating facilitating actions and tangible results in the implementation of psychological interventions for depression? In clinical psychology, research seems to focus on carrying out efficacy studies and randomized controlled trials, where high levels of rigor are established by exerting control over the environment and possible influencing factors. However, this rigor has a very high cost for the population: the difficulty of transferring psychological practices to daily clinical practice, thus perpetuating the time-lapse between science and practice [9,30,31]. As a result, clinical psychology is generating scientific quality in terms of controlled efficacy, but external validity is not being considered [89]. One of the limitations we have identified has been the challenge of classifying study designs. To address this issue in future research, we recommend advocating for the establishment of a standardized nomenclature for various study designs within the domain of IR, encompassing hybrid designs as well. Hybrid efficacy–implementation studies are presented to address the translational problem, offering bridges between science and clinical settings [90,91]. In the classic scientific process, the effectiveness of the treatment would be established first, and then the effectiveness of the implementation. However, this produces resistance from the scientific community in relation to time and investment costs. Consequently, hybrid studies propose a more direct and rapid approach to assess the effectiveness of interventions and their implementation [41,84]. In this regard, hybrid studies are established as a promising methodology to position EBPP in IR.

While this review provides a valuable perspective on studies that implement EBPPs to address depression, it is essential to note that studies solely focused on assessing barriers and strategies or those that exclusively enumerate implementation strategies have not been incorporated. It would be worthwhile for future research to encompass these two categories of studies to further enhance our understanding of crucial aspects related to addressing depression in real-world settings [37].

## 5. Conclusions

IR faces several challenges, including the need for a specific theoretical framework, terminology issues, a lack of specific reporting guidelines, and the need for a broader exploration of implementation outcomes. Within the field of clinical psychology, especially in the context of addressing depressive disorders, our review has revealed a relatively low volume of implementation studies of EBPPs. Despite the limited number of studies, there is reason for optimism given the substantial number of implementation study protocols, signaling a growing interest in psychology.

The studies we examined exhibited shortcomings in terms of implementation quality. Nevertheless, the high quality of the EBPPs being implemented and their suitability for addressing mental health disorders highlight that IR in clinical psychology is striving for excellence. As the quality of care for psychological issues in everyday clinical settings relies on insights from implementation studies, it is conceivable that clinical psychology may increasingly embrace IR in the near future.

This review offers an overview of the current state of IR within clinical psychology and the implementation of EBPP. Future research endeavors should focus on establishing a consistent theoretical framework, fostering the growth of IR in psychology, and advancing research and strategies for implementing EBPP within clinical settings.

## Figures and Tables

**Table 1:** Goals and units of analysis of the studies.

Study	Goal of the Study	Units of Analysis
Asrat et al., 2021 [46]	To assess the acceptability and feasibility of peer-administered group IPT for depressive symptoms among people living with HIV/AIDS in Northwest Ethiopia.	Peer-counsellors, supervisors, and patients
Bina et al., 2017 [47]	To examine social workers’ perspectives of provider- and organization-related barriers to implementing IPTin a primary care setting in Israel for women who have postpartum depression symptoms.	Social workers
Bloomquist et al., 2017 [48]	To describe the Health Emotions Programs and Behaviour Development Program to address depression and behavior problems of adolescents demonstrating how they were brought into a community mental health setting and evaluated for their effects on youth and family outcomes.	Patients, practitioners, parents
Chen et al., 2019 [49]	Describe and assess feasibility and implementation process of Dynamic Interpersonal Therapy to address Veterans Affairs depression in medical center considering providers, patients, and systems barriers.	Patients, Therapists
Clignet et al., 2016 [50]	To explore the nurses’ perceptions of the barriers and facilitators in the implementation of an intervention Systematic Activation Method to address late-life depression in mental health nursing care.	Nurses, patients
Dear et al., 2020 [51]	Assess the acceptability and effectiveness of an internet-delivered and therapist-guided intervention, UniWellbeing, to address anxiety and depression, when delivered as routine care for students attending a university counselling service.	Patients
Drozd et al., 2018 [52]	To assess training and implementation of an internet intervention (MammaMia) to address perinatal depression in Norwegian well-baby clinics examining implementation variables, barriers, and facilitators.	Health and administrative staff and organization
Eiraldi et al., 2019 [53]	To describe the fidelity, perceived acceptability, and student outcomes of a group CBT in schools	Students, parents and school staff
Fortney et al., 2012 [54]	To evaluate the feasibility of evidence-based quality improvement as a strategy to facilitate the adoption of Collaborative-care management in Community-Based outpatient Clinics to address Veterans’ Affairs depression.	Organization, staff, patients
Fuchs et al., 2016 [55]	To evaluate the implementation of an acceptance and mindfulness-based group for primary care patients with depression and anxiety considering the group’s feasibility, acceptability, penetration, and sustainability, and provide initial outcome data.	Patients
Geraedts et al., 2014 [56]	To assess the feasibility of the intervention Happy@Work and explore possible barriers and facilitators for future implementation of the intervention into routine practice.	Patients and intervention providers and organization
Hadjistavropoulos et al., 2017 [57]	To assess and identify barriers and facilitators that influenced Internet-delivered CBT implementation in community mental health clinics distributed across one province.	Therapist and managers
Israel et al., 2013 [58]	To assess the feasibility of the intervention Attachment-Based Family Therapy to address depression in a hospital-based public mental health clinic, select regular staff therapists for training and test the intervention effectiveness.	Patients and therapists and institution
Johnson et al., 2020 [59]	To assess potentially influencing factors in the implementation of IPT for depression in prisons considering feasibility and acceptability.	Patients, providers, and administrators
Kanine et al., 2021 [60]	To assess fidelity, feasibility, and acceptability of delivering IPT-AST to adolescents from marginalized backgrounds within urban PC.	Patients, caregivers, and supervisor.
Karlin et al., 2019 [61]	To train professionals in CBT-D and examine initial feasibility and effectiveness of individualized training in and implementation of the intervention.	Patients and therapists.
Kramer et al., 2008 [62]	To assess the implementation of CBT for depressed adolescents seeking public sector mental health services, focusing on the extent it was implemented in two publicly funded mental healthcare clinics and the process and the factors influencing it.	Patients, therapists, and organization
Lindholm et al., 2019 [63]	Assess the quantitative reach of the EBT and the explanatory factors considering the therapists’ views on the usefulness of BA on addressing depression.	Therapists
Lusk et al., 2011 [64]	To promote the implementation of the COPE program to adolescents experiencing depressive symptoms and to determine feasibility and efficacy with this population.	Patients, psychiatric nurses, and parents
MacPherson et al., 2014 [65]	To assess descriptive and quantitative data on the implementation of MF-PEP at two outpatient community clinics for children with mood disorders and their parents using Proctor implementation outcome taxonomy.	Parents, children, MF-PEP therapists, referring clinicians and agency-level observations.
Mignogna et al., 2014 [66]	To assess feasibility and acceptability of a multifaceted implementation strategies in the implementation of a bCBT to address depression and/or anxiety in PC considering preliminary fidelity and adoption measures.	Clinicians
Mignogna et al., 2018 [67]	To assess provider’s perspectives on fidelity of bCBT implemented in PC settings.	Providers
Morrison et al., 2014 [68]	To explore the Type 2 translation gap by conducting an implementation pilot of MindBalance, a web-based intervention for depression in three IAPT services.	Patients
Parhiala et al., 2019 [69]	To assess the effectiveness, feasibility, and acceptability of IPC as compared with BPS in Finnish schools.	Patients and counsellors
Peterson et al., 2018 [70]	To evaluate the implementation strategies used and feasibility of implementing CETA in Washington State PBH.	Clinicians and patients
Rasmussen et al., 2019 [71]	To identify barriers and facilitators in the active phase of the EPIS model in the implementation of the EMOTION program within the group leaders’ organizational context.	Group leaders and organization
Santucci et al., 2014 [72]	To assess feasibility, acceptability and effectiveness of the implementation of the program BtB to address depression and anxiety in a university-based health setting.	Patients
Sit et al., 2022 [73]	To assess the feasibility and preliminary effectiveness of delivering Step-by-Step in a University setting for Chinese young adults with minimal peer-support guidance model to address depression and anxiety.	Patients
Steinfeld et al., 2009 [74]	To describe the experience of psychologists who designed and implemented an intensive training program to diffuse one CBT for the treatment of anxiety and depression in a large mental health services delivery system.	Mental health providers and organization
Walser et al., 2013 [75]	To assess the training of mental health clinicians in ACT-D considering the impact of the implementation in professionals and the patients.	Therapist and patients
Wilfley et al., 2020 [76]	To compare two methods of training in IPT to address university students with depression and/or eating disorders.	Therapists

ACT-D = Acceptance and Commitment Therapy for Depression; BA = Behavioral Activation; bCBT = Brief Cognitive Behavioral Therapy; BPS = Brief Psychosocial Support; BtB = Beating the Blues; CBT = Cognitive Behavioral Therapy; CETA = Common Elements Treatment Approach; COPE = Creating Opportunities for Personal Empowerment; EBT = Evidence Based Treatment; EPIS: Exploration, Preparation, Implementation and Sustainment; HIV/AIDS = Human Immunodeficiency Virus/Acquired Immunodeficiency Syndrome; IPC = Interpersonal Counseling; IPT-AST = Inter-Personal Therapy-Adolescents Skill Training; IPT = Inter-Personal Therapy; MF-PEP = Multi-Family Psychoeducational Psychotherapy; PBH = Public Behavioral Health; PC = Primary Care.

## Supplementary Materials

The following supporting information can be downloaded at: https://www.mdpi.com/article/10.3390/jcm14176347/s1, Supplementary File S1: PRISMA 2020 Checklist; File S2: Search strategy; Table S1: Characteristics of the studies; Table S2: Assessment according to the CFIR framework for study implementation.

## Abbreviations

ARC	Availability, Responsiveness and Continuity
BA	Behavioral Activation
BDIT	Brief Dynamic Interpersonal Therapy
BPS	Brief Psychosocial Support
CB	Cognitive Behavioral
CBT	Cognitive-Behavioral Therapy
CCM	Collaborative Care Management
CETA	Common Elements Treatment Approach
CFIR	Consolidated Framework for Implementation Research
COPE	Creating Opportunities for Personal Empowerment
DBT	Dialectical Behavioral Therapy
EBPP	Evidence-Based Psychological Practice
EPIS	Exploration, Preparation/Adoption, Implementation and Sustainment
FOCUS-PDSA	Find, Organize, Clarify, Understand, Select—Plan, Do, Study, Act
IPT-AST	IPT-Adolescents Skills Training
IPT	Inter-Personal Therapy
IQA	Intensive Quality Assurance
IR	Implementation Research
MF-PEP	Multi-Family Psychoeducational Psychotherapy
NICE	National Institute for Health and Care Excellence
PARISH	Promoting Action on Research Implementation in Health Services
PICO	Participants, Interventions, Comparisons and Outcomes
PRISMA	Preferred Reporting Items for Systematic Reviews and Meta-Analyses
PTSD	Post-Traumatic Stress Disorder
RE-AIM	Reach, Effectiveness, Adoption, Implementation, and Maintenance
SAM	System Activation Method
US	United States
VA	Veterans Affairs
WHO	World Health Organization
YLDs	Years Lived with Disability
