# Supplementary material for: Implementation of Evidence-Based Psychological Treatments to Address Depressive Disorders: A Systematic Review
Source: J Clin Med. 2025 Sep 8;14(17):6347. doi: 10.3390/jcm14176347 (PMC12429828; doi:10.3390/jcm14176347)
Supplement: Supplementary file 1 [file jcm-14-06347-s001.zip › Supplementary Materials_File S2.pdf]

Supplementary File S2. Search strategy

The advanced search strategies are usually listed in an appendix. The article contains a short description of the method and PRISMA flowchart. Please note that the numbers stated in the flow chart correspond to those of the written search strategies.

**Database searched**	**Platform**	**Years of coverage**	**Records**	**Records after duplicates removed**
PubMed	PubMed	1946 – Present	1.619	1.618
Embase	Embase.com	1971 - Present	1.425	404
APA PsycInfo	EBSCO	1806 - Present	831	221
Web of Science Core Collection*	Clarivate	1975 - Present	1.856	693
Scopus	Elsevier	1823 - Present	1.758	231
Cochrane Database of Systematic Reviews + CENTRAL	Wiley	1992 - Present	1.308	590
**Total**			**8.797**	**3.757**

*Science Citation Index Expanded (1975-present) ; Social Sciences Citation Index (1975-present) ; Arts & Humanities Citation Index (1975-present) ; Conference Proceedings Citation Index- Science (1990-present) ; Conference Proceedings Citation Index- Social Science & Humanities (1990-present) ; Emerging Sources Citation Index (2005-present)

	PubMed	
Search	Query	Results
#4	Search: #1 AND #2 AND #3 Sort by: Most Recent	1,619
#3	Search: "Health Plan implementation"[Mesh] OR "Feasibility Studies"[Mesh] OR implement[ti] OR implement[ot] OR implementation[ti] OR implementation[ot] OR implemented[ti] OR implemented[ot] OR implementing[ti] OR implementing[ot] OR normalisation[ti] OR normalisation[ot] OR normalise[ti] OR normalise[ot] OR normalised[ti] OR normalised[ot] OR normalising[ti] OR normalising[ot] OR normalization[ti] OR normalization[ot] OR normalize[ti] OR normalize[ot] OR normalized[ti] OR normalized[ot] OR normalizing[ti] OR normalizing[ot] OR sustain[ti] OR sustain[ot] OR sustainability[ti] OR sustainability[ot] OR sustainable[ti] OR sustainable[ot] OR sustained[ti] OR sustained[ot] OR acceptability[ti] OR acceptability[ot] OR adoption[ti] OR adoption[ot] OR appropriateness[ti] OR appropriateness[ot] OR fidelity[ti] OR fidelity[ot] OR coverage[ti] OR coverage[ot] OR sustainability[ti] OR sustainability[ot] OR feasibility[ti] OR feasibility[ot] Sort by: Most Recent	278,298
#2	Search: "Psychotherapy"[Mesh] OR psychotherap*[Tiab] OR cbt[Tiab] OR mindfulness[Tiab] OR "cognitive restructuring"[Tiab] OR "behavior therap*"[Tiab] OR "behaviors therap*"[Tiab] OR "behavioral therap*"[Tiab] OR "behaviour therap*"[Tiab] OR "behaviours therap*"[Tiab] OR "behavioural therap*"[Tiab] OR "cognition therap*"[Tiab] OR "cognitive therap*"[tiab] OR "behavior treatment*"[Tiab] OR "behaviors treatment*"[Tiab] OR "behavioral treatment*"[Tiab] OR "behaviour treatment*"[Tiab] OR "behaviours treatment*"[Tiab] OR "behavioural treatment*"[Tiab] OR "cognition treatment*"[Tiab] OR "cognitive treatment*"[tiab] OR "behavior intervention*"[Tiab] OR "behaviors intervention*"[Tiab] OR "behavioral intervention*"[Tiab] OR "behaviour intervention*"[Tiab] OR "behaviours intervention*"[Tiab] OR "behavioural intervention*"[Tiab] OR "cognition intervention*"[Tiab] OR "cognitive intervention*"[tiab] OR	272,928

	PubMed	
Search	Query	Results
	"behavior activation*"[Tiab] OR "behaviors activation*"[Tiab] OR "behavioral activation*"[Tiab] OR "behaviour activation*"[Tiab] OR "behaviours activation*"[Tiab] OR "behavioural activation*"[Tiab] OR "exposure therap*"[tiab] OR "psychological intervention*"[tiab] OR "interpersonal therapy" [Tiab] OR ipt[Tiab] OR "short-term-psychodynamic psychotherapy" [Tiab] OR stpp[Tiab] OR "individual problem-solving"[Tiab] OR "problem-solving therapy"[Tiab] OR pst[Tiab] OR "mindfulness-based cognitive therapy"[Tiab] OR mcbt[Tiab] OR "psychodynamic"[Tiab] OR psychoeducation[Tiab] OR "Acceptance and commitment*"[tiab] OR "Dialectical behaviour therapy"[Tiab] OR dbt[tiab] OR "Emotion-focused therapy"[Tiab] OR eft[tiab] OR "Eye movement desensitisation and reprocessing"[Tiab] OR "Eye Movement Desensitization Reprocessing"[tiab] OR "Eye Movement Desensitization and Reprocessing"[tiab] OR "Eye Movement Desensitisation Reprocessing"[tiab] OR "Eye Movement Desensitisation and Reprocessing"[tiab] OR EMDR[tiab] OR "Family intervention*"[Tiab] OR "schema therapy"[Tiab] OR "Solution-focused therapy"[Tiab] Sort by: Most Recent	
#1	Search: "Depressive Disorder"[Mesh] OR "major depression*"[tiab] OR "depressive disorder*"[tiab] OR "severe depression*"[tiab] OR MDD[tiab] OR "recurrent depression*"[tiab] OR "remitted depression*"[tiab] OR "depressive episod*"[tiab] OR "chronic depression*"[tiab] OR "treatment resistant depression*"[tiab] OR "persistent depression*"[tiab] OR dysthymi*[tiab] OR depress*[tiab] Sort by: Most Recent	539,745

Key: [Mesh]= medical subject heading, [tiab] = title, abstract, author supplied keywords, [ti] = title, [ot] = author supplied keywords

	**Embase (Embase.com)**	
**No.**	**Query**	**Results**
**#5**	#4 AND (**'article'**/it OR **'article in press'**/it OR **'conference paper'**/it OR **'editorial'**/it OR **'erratum'**/it OR **'letter'**/it OR **'note'**/it OR **'review'**/it OR **'short survey'**/it)	**1,425**
**#4**	**#1** AND **#2** AND **#3**	**1,697**
**#3**	**'implementation science'**/exp OR **'implementation'**/exp OR **'health care planning'**/exp OR **implement**:ti,kw OR **implementation**:ti,kw OR **implemented**:ti,kw OR **implementing**:ti,kw OR **normalisation**:ti,kw OR **normalise**:ti,kw OR **normalised**:ti,kw OR **normalising**:ti,kw OR **normalization**:ti,kw OR **normalize**:ti,kw OR **normalized**:ti,kw OR **normalizing**:ti,kw OR **sustain**:ti,kw OR **sustainable**:ti,kw OR **sustained**:ti,kw OR **acceptability**:ti,kw OR **adoption**:ti,kw OR **appropriateness**:ti,kw OR **fidelity**:ti,kw OR **coverage**:ti,kw OR **sustainability**:ti,kw OR **'feasibility'**:ti,kw	**393,484**
**#2**	**'psychotherapy'**/exp OR **psychotherap***:ab,ti,kw OR **cbt**:ab,ti,kw OR **mindfulness**:ab,ti,kw OR **'cognitive restructuring'**:ab,ti,kw OR **'behavior therap*'**:ab,ti,kw OR **'behaviors therap*'**:ab,ti,kw OR **'behavioral therap*'**:ab,ti,kw OR **'behaviour therap*'**:ab,ti,kw OR **'behaviours therap*'**:ab,ti,kw OR **'behavioural therap*'**:ab,ti,kw OR **'cognition therap*'**:ab,ti,kw OR **'cognitive therap*'**:ab,ti,kw OR **'behavior treatment*'**:ab,ti,kw OR **'behaviors treatment*'**:ab,ti,kw OR **'behavioral treatment*'**:ab,ti,kw OR **'behaviour treatment*'**:ab,ti,kw OR **'behaviours treatment*'**:ab,ti,kw OR **'behavioural treatment*'**:ab,ti,kw OR **'cognition treatment*'**:ab,ti,kw OR **'cognitive treatment*'**:ab,ti,kw OR **'behavior intervention*'**:ab,ti,kw OR **'behaviors intervention*'**:ab,ti,kw OR **'behavioral intervention*'**:ab,ti,kw OR **'behaviour intervention*'**:ab,ti,kw OR **'behaviours intervention*'**:ab,ti,kw OR **'behavioural**	**362,235**

	**intervention*'**:ab,ti,kw OR **'cognition intervention*'**:ab,ti,kw OR **'cognitive intervention*'**:ab,ti,kw OR **'behavior activation*'**:ab,ti,kw OR **'behaviors activation*'**:ab,ti,kw OR **'behavioral activation*'**:ab,ti,kw OR **'behaviour activation*'**:ab,ti,kw OR **'behaviours activation*'**:ab,ti,kw OR **'behavioural activation*'**:ab,ti,kw OR **'exposure therap*'**:ab,ti,kw OR **'psychological intervention*'**:ab,ti,kw OR **'interpersonal therapy'**:ab,ti,kw OR **ipt**:ab,ti,kw OR **'short-term-psychodynamic psychotherapy'**:ab,ti,kw OR **stpp**:ab,ti,kw OR **'individual problem-solving'**:ab,ti,kw OR **'problem-solving therapy'**:ab,ti,kw OR **pst**:ab,ti,kw OR **'mindfulness-based cognitive therapy'**:ab,ti,kw OR **mcbt**:ab,ti,kw OR **'psychodynamic'**:ab,ti,kw OR **psychoeducation**:ab,ti,kw OR **'acceptance and commitment*'**:ab,ti,kw OR **'dialectical behaviour therapy'**:ab,ti,kw OR **dbt**:ab,ti,kw OR **'emotion-focused therapy'**:ab,ti,kw OR **eft**:ab,ti,kw OR **'eye movement desensitization reprocessing'**:ab,ti,kw OR **'eye movement desensitization and reprocessing'**:ab,ti,kw OR **'eye movement desensitisation reprocessing'**:ab,ti,kw OR **'eye movement desensitisation and reprocessing'**:ab,ti,kw OR **emdr**:ab,ti,kw OR **'family intervention'**:ab,ti,kw OR **'schema therapy'**:ab,ti,kw OR **'solution-focused therapy'**:ab,ti,kw	
**#1**	**'major depression'**/exp OR **'major depression*'**:ab,ti,kw OR **'depressive disorder*'**:ab,ti,kw OR **'severe depression*'**:ab,ti,kw OR **mdd**:ab,ti,kw OR **'recurrent depression*'**:ab,ti,kw OR **'remitted depression*'**:ab,ti,kw OR **'depressive episod*'**:ab,ti,kw OR **'chronic depression*'**:ab,ti,kw OR **'treatment resistant depression*'**:ab,ti,kw OR **'persistent depression*'**:ab,ti,kw OR **dysthymi***:ab,ti,kw OR **depress***:ab,ti,kw	**718,837**

Key: Ab, ti, kw searches in abstract, title and author supplied keywords, /exp searches Emtree preferred indexing term, it=publication type

	APA PsycInfo (EBSCO)		
#	Query	Limiters/Expanders	Results
S5	S1 AND S2 AND S3	Limiters – Academic journals	831
S4	S1 AND S2 AND S3	Search modes - Boolean/Phrase	920
S3	TI(implement OR implementation OR implemented OR implementing OR normalisation OR normalise OR normalised OR normalising OR normalization OR normalize OR normalized OR normalizing OR sustain OR sustainability OR sustainable OR sustained OR acceptability OR adoption OR appropriateness OR fidelity OR coverage OR sustainability OR feasibility) OR KW(implement OR implementation OR implemented OR implementing OR normalisation OR normalise OR normalised OR normalising OR normalization OR normalize OR normalized OR normalizing OR sustain OR sustainability OR sustainable OR sustained OR acceptability OR adoption OR appropriateness OR fidelity OR coverage OR sustainability OR feasibility)	Search modes - Boolean/Phrase	69,240
S2	DE "Cognitive Behavior Therapy" OR DE "Exposure Therapy" OR DE "Behavior Therapy" OR DE "Imaginal Exposure" OR DE "Implosive Therapy" OR DE "In Vivo Exposure" OR DE "Prolonged Exposure Therapy" OR DE "Systematic Desensitization Therapy" OR DE "Virtual Reality Exposure Therapy" OR DE	Search modes - Boolean/Phrase	269,440

	"Acceptance and Commitment Therapy" OR DE "Dialectical Behavior Therapy" OR DE "Mindfulness-Based Interventions" OR DE "Mindfulness" OR DE "Schema Therapy" OR DE "Psychotherapy" OR DE "Brief Psychotherapy" OR DE "Emotion Focused Therapy" OR DE "Eye Movement Desensitization Therapy" OR DE "Individual Psychotherapy" OR DE "Integrative Psychotherapy" OR DE "Interpersonal Psychotherapy" OR DE "Psychodynamic Psychotherapy" OR DE "Solution Focused Therapy" OR DE "Cognitive Therapy" OR DE "Online Therapy" OR TI(psychotherap* OR cbt OR mindfulness OR "cognitive restructuring" OR "behavior therap*" OR "behaviors therap*" OR "behavioral therap*" OR "behaviour therap*" OR "behaviours therap*" OR "behavioural therap*" OR "cognition therap*" OR "cognitive therap*" OR "behavior treatment*" OR "behaviors treatment*" OR "behavioral treatment*" OR "behaviour treatment*" OR "behaviours treatment*" OR "behavioural treatment*" OR "cognition treatment*" OR "cognitive treatment*" OR "behavior intervention*" OR "behaviors intervention*" OR "behavioral intervention*" OR "behaviour intervention*" OR "behaviours intervention*" OR "behavioural intervention*" OR "cognition intervention*" OR "cognitive intervention*" OR "behavior activation*" OR "behaviors activation*" OR "behavioral activation*" OR "behaviour activation*" OR "behaviours activation*" OR "behavioural activation*" OR "exposure therap*" OR "psychological intervention*" OR "interpersonal therapy" OR ipt OR "short-term-psychodynamic psychotherapy" OR stpp OR "individual problem-solving" OR "problem-solving therapy" OR pst OR "mindfulness-based cognitive therapy" OR mcbt OR "psychodynamic" OR psychoeducation OR "Acceptance and commitment*" OR "Dialectical behaviour therapy" OR dbt OR "Emotion-focused therapy" OR eft OR "Eye movement desensitisation and reprocessing" OR "Eye Movement Desensitization Reprocessing" OR "Eye Movement Desensitization and Reprocessing" OR "Eye Movement Desensitisation Reprocessing" OR "Eye Movement Desensitisation and Reprocessing" OR EMDR OR "Family intervention" OR "schema therapy" OR "Solution-focused therapy") OR AB(psychotherap* OR cbt OR mindfulness OR "cognitive restructuring" OR "behavior therap*" OR "behaviors therap*" OR "behavioral therap*" OR "behaviour therap*" OR "behaviours therap*" OR "behavioural therap*" OR "cognition therap*" OR "cognitive therap*" OR "behavior treatment*" OR "behaviors treatment*" OR "behavioral treatment*" OR "behaviour treatment*" OR "behaviours treatment*" OR "behavioural treatment*" OR "cognition treatment*" OR "cognitive treatment*" OR "behavior intervention*" OR "behaviors intervention*" OR "behavioral intervention*" OR "behaviour intervention*" OR "behaviours intervention*" OR "behavioural intervention*" OR "cognition intervention*" OR "cognitive intervention*" OR "behavior activation*" OR "behaviors activation*" OR "behavioral activation*" OR "behaviour activation*" OR "behaviours activation*" OR "behavioural activation*" OR "exposure therap*" OR "psychological intervention*" OR "interpersonal therapy" OR ipt OR "short-term-psychodynamic psychotherapy" OR stpp OR "individual problem-solving" OR "problem-solving therapy" OR pst OR "mindfulness-based cognitive therapy" OR mcbt OR "psychodynamic" OR psychoeducation OR "Acceptance and commitment*" OR "Dialectical behaviour therapy" OR dbt OR "Emotion-focused therapy" OR eft OR "Eye movement desensitisation and reprocessing" OR "Eye Movement Desensitization Reprocessing" OR "Eye Movement Desensitization and Reprocessing" OR "Eye Movement Desensitisation Reprocessing" OR "Eye Movement Desensitisation and Reprocessing" OR EMDR OR "Family intervention" OR "schema therapy" OR "Solution-focused therapy") OR KW(psychotherap* OR cbt OR mindfulness OR "cognitive restructuring" OR "behavior therap*" OR "behaviors therap*" OR "behavioral therap*" OR "behaviour therap*" OR "behaviours therap*" OR "behavioural therap*" OR "cognition therap*" OR "cognitive therap*" OR "behavior treatment*" OR "behaviors treatment*" OR "behavioral treatment*" OR "behaviour treatment*" OR "behaviours treatment*" OR "behavioural treatment*" OR "cognition treatment*" OR "cognitive treatment*" OR "behavior intervention*" OR "behaviors intervention*" OR "behavioral intervention*" OR "behaviour intervention*" OR "behaviours intervention*" OR "behavioural intervention*" OR "cognition intervention*" OR "cognitive intervention*" OR "behavior activation*"		

	OR "behaviors activation*" OR "behavioral activation*" OR "behaviour activation*" OR "behaviours activation*" OR "behavioural activation*" OR "exposure therap*" OR "psychological intervention*" OR "interpersonal therapy" OR ipt OR "short-term-psychodynamic psychotherapy" OR stpp OR "individual problem-solving" OR "problem-solving therapy" OR pst OR "mindfulness-based cognitive therapy" OR mcbt OR "psychodynamic" OR psychoeducation OR "Acceptance and commitment*" OR "Dialectical behaviour therapy" OR dbt OR "Emotion-focused therapy" OR eft OR "Eye movement desensitisation and reprocessing" OR "Eye Movement Desensitization Reprocessing" OR "Eye Movement Desensitization and Reprocessing" OR "Eye Movement Desensitisation Reprocessing" OR "Eye Movement Desensitisation and Reprocessing" OR EMDR OR "Family intervention" OR "schema therapy" OR "Solution-focused therapy")		
S1	DE “Major Depression" OR DE "Anaclitic Depression" OR DE "Dysthymic Disorder" OR DE "Endogenous Depression" OR DE "Late Life Depression" OR DE "Postpartum Depression" OR DE "Reactive Depression" OR DE "Recurrent Depression" OR DE "Treatment Resistant Depression" OR TI("major depression*" OR "depressive disorder*" OR "severe depression*" OR MDD OR "recurrent depression*" OR "remitted depression*" OR "depressive episod*" OR "chronic depression*" OR "treatment resistant depression*" OR "persistent depression*" OR dysthymi* OR depress*) OR AB("major depression*" OR "depressive disorder*" OR "severe depression*" OR MDD OR "recurrent depression*" OR "remitted depression*" OR "depressive episod*" OR "chronic depression*" OR "treatment resistant depression*" OR "persistent depression*" OR dysthymi* OR depress*) OR KW("major depression*" OR "depressive disorder*" OR "severe depression*" OR MDD OR "recurrent depression*" OR "remitted depression*" OR "depressive episod*" OR "chronic depression*" OR "treatment resistant depression*" OR "persistent depression*" OR dysthymi* OR depress*)	Search modes - Boolean/Phrase	338,557

Key: DE= Descriptors,TI= title, AB= abstract and KW= Searches for keywords in the uncontrolled content description of the document

	Web of Science (Core collection) - Clarivate	
Nr.	Query	Results
#6	#5 AND #2 AND #1	1,856
#5	#4 OR #3	647,095
#4	AK=(implement OR implementation OR implemented OR implementing OR normalisation OR normalise OR normalised OR normalising OR normalization OR normalize OR normalized OR normalizing OR sustain OR sustainability OR sustainable OR sustained OR acceptability OR adoption OR appropriateness OR fidelity OR coverage OR sustainability OR feasibility)	231,097
#3	TI=(implement OR implementation OR implemented OR implementing OR normalisation OR normalise OR normalised OR normalising OR normalization OR normalize OR normalized OR normalizing OR sustain OR sustainability OR sustainable OR sustained OR acceptability OR adoption OR appropriateness OR fidelity OR coverage OR sustainability OR feasibility)	524,234
#2	TS=(psychotherap* OR cbt OR mindfulness OR "cognitive restructuring" OR "behavior therap*" OR "behaviors therap*" OR "behavioral therap*" OR "behaviour therap*" OR "behaviours therap*" OR "behavioural therap*" OR "cognition therap*" OR "cognitive therap*" OR "behavior treatment*" OR "behaviors treatment*" OR "behavioral treatment*" OR "behaviour treatment*" OR "behaviours treatment*" OR "behavioural treatment*" OR "cognition treatment*" OR "cognitive treatment*" OR "behavior intervention*" OR "behaviors intervention*" OR "behavioral intervention*" OR "behaviour intervention*" OR "behaviours intervention*" OR "behavioural intervention*" OR "cognition intervention*" OR "cognitive intervention*" OR "behavior activation*" OR "behaviors activation*" OR "behavioral activation*" OR "behaviour activation*" OR "behaviours activation*" OR "behavioural activation*" OR "exposure therap*" OR "psychological intervention*" OR "interpersonal therapy" OR ipt OR "short-term-	220,565

	psychodynamic psychotherapy" OR stpp OR "individual problem-solving" OR "problem-solving therapy" OR pst OR "mindfulness-based cognitive therapy" OR mcbt OR "psychodynamic" OR psychoeducation OR "Acceptance and commitment*" OR "Dialectical behaviour therapy" OR dbt OR "Emotion-focused therapy" OR eft OR "Eye movement desensitisation and reprocessing" OR "Eye Movement Desensitization Reprocessing" OR "Eye Movement Desensitization and Reprocessing" OR "Eye Movement Desensitisation Reprocessing" OR "Eye Movement Desensitisation and Reprocessing" OR EMDR OR "Family intervention" OR "schema therapy" OR "Solution-focused therapy")	
#1	TS=("major depression*" OR "depressive disorder*" OR "severe depression*" OR MDD OR "recurrent depression*" OR "remitted depression*" OR "depressive episod*" OR "chronic depression*" OR "treatment resistant depression*" OR "persistent depression*" OR dysthymi* OR depress*)	724,581

Key: TS = topic, which includes title, abstract, author keywords and Web of Science Keywords Plus./ TI = title, AK=author keywords

	Scopus (Elsevier)	
History Count	Search Terms	Results
6	#1 AND #2 AND #5	1,758
5	#3 OR #4	937,742
4	AUTHKEY (implement OR implementation OR implemented OR implementing OR normalisation OR normalise OR normalised OR normalising OR normalization OR normalize OR normalized OR normalizing OR sustain OR sustainability OR sustainable OR sustained OR acceptability OR adoption OR appropriateness OR fidelity OR coverage OR sustainability OR feasibility)	336,453
3	TITLE (implement OR implementation OR implemented OR implementing OR normalisation OR normalise OR normalised OR normalising OR normalization OR normalize OR normalized OR normalizing OR sustain OR sustainability OR sustainable OR sustained OR acceptability OR adoption OR appropriateness OR fidelity OR coverage OR sustainability OR feasibility)	760,248
2	TITLE-ABS-KEY (psychotherap* OR cbt OR mindfulness OR "cognitive restructuring" OR "behavior therap*" OR "behaviors therap*" OR "behavioral therap*" OR "behaviour therap*" OR "behaviours therap*" OR "behavioural therap*" OR "cognition therap*" OR "cognitive therap*" OR "behavior treatment*" OR "behaviors treatment*" OR "behavioral treatment*" OR "behaviour treatment*" OR "behaviours treatment*" OR "behavioural treatment*" OR "cognition treatment*" OR "cognitive treatment*" OR "behavior intervention*" OR "behaviors intervention*" OR "behavioral intervention*" OR "behaviour intervention*" OR "behaviours intervention*" OR "behavioural intervention*" OR "cognition intervention*" OR "cognitive intervention*" OR "behavior activation*" OR "behaviors activation*" OR "behavioral activation*" OR "behaviour activation*" OR "behaviours activation*" OR "behavioural activation*" OR "exposure therap*" OR "psychological intervention*" OR "interpersonal therapy" OR ipt OR "short-term-psychodynamic psychotherapy" OR stpp OR "individual problem-solving" OR "problem-solving therapy" OR pst OR "mindfulness-based cognitive therapy" OR mcbt OR "psychodynamic" OR psychoeducation OR "Acceptance and commitment*" OR "Dialectical behaviour therapy" OR dbt OR "Emotion-focused therapy" OR eft OR "Eye movement desensitisation and reprocessing" OR "Eye Movement Desensitization Reprocessing" OR "Eye Movement Desensitization and Reprocessing" OR "Eye Movement Desensitisation Reprocessing" OR "Eye Movement Desensitisation and Reprocessing" OR emdr OR "Family intervention" OR "schema therapy" OR "Solution-focused therapy") ...View More	358,013
1	TITLE-ABS-KEY ("major depression*" OR "depressive disorder*" OR "severe depression*" OR mdd OR "recurrent depression*" OR "remitted depression*" OR "depressive episod*" OR "chronic depression*" OR "treatment resistant depression*" OR "persistent depression*" OR dysthymi* OR depress*)	985,361

Key: ABS, TITLE, AUTHKEY searches in abstract, title and author supplied keywords

	Cochrane Database of Systematic Reviews + CENTRAL (Wiley)	

ID	Search	Hits
#6	#4 AND CENTRAL	1307
#5	#4 AND CDSR	1
#4	#1 AND #2 AND #3	1308
#3	(implement OR implementation OR implemented OR implementing OR normalisation OR normalise OR normalised OR normalising OR normalization OR normalize OR normalized OR normalizing OR sustain OR sustainability OR sustainable OR sustained OR acceptability OR adoption OR appropriateness OR fidelity OR coverage OR sustainability OR feasibility):ti,kw	44002
#2	(psychotherap* OR cbt OR mindfulness OR "cognitive restructuring" OR "behavior therapy" OR “behavior therapies” OR "behaviors therapy" OR “behaviors therapies” OR "behavioral therapy" OR “behavioral therapies” OR "behaviour therapy" OR “behaviour therapies” OR "behaviours therapy" OR “behaviours therapies” OR "behavioural therapy" OR “behavioural therapies” OR "cognition therapy" OR “cognition therapies” OR "cognitive therapy" OR “cognitive therapies” OR "behavior treatment" OR "behaviors treatment" OR "behavioral treatment" OR "behaviour treatment" OR "behaviours treatment" OR "behavioural treatment" OR "cognition treatment" OR "cognitive treatment" OR "behavior intervention" OR “behavior interventions” OR "behaviors intervention" OR “behaviors interventions” OR "behavioral intervention" OR “behavioral interventions” OR "behaviour intervention" OR “behaviour interventions” OR "behaviours intervention" OR “behaviours interventions” OR "behavioural intervention" OR “behavioural interventions” OR "cognition intervention" OR “cognition interventions” OR "cognitive intervention" OR “cognitive interventions” OR "behavior activation" OR "behaviors activation" OR "behavioral activation" OR "behaviour activation" OR "behaviours activation" OR "behavioural activation" OR "exposure therapy" OR “exposure therapies” OR "psychological intervention" OR “psychological interventions” OR "interpersonal therapy" OR ipt OR "short-term-psychodynamic psychotherapy" OR stpp OR "individual problem-solving" OR "problem-solving therapy" OR pst OR "mindfulness-based cognitive therapy" OR mcbt OR "psychodynamic" OR psychoeducation OR "Acceptance and commitment" OR "Dialectical behaviour therapy" OR dbt OR "Emotion-focused therapy" OR eft OR "Eye movement desensitisation and reprocessing" OR "Eye Movement Desensitization Reprocessing" OR "Eye Movement Desensitization and Reprocessing" OR "Eye Movement Desensitisation Reprocessing" OR "Eye Movement Desensitisation and Reprocessing" OR EMDR OR "Family intervention" OR "schema therapy" OR "Solution-focused therapy"):ti,ab,kw	57393
#1	("major depression" OR "depressive disorder" OR “depressive disorders” OR "severe depression*" OR MDD OR "recurrent depression" OR "remitted depression" OR "depressive episode" OR “depressive episodes” OR "chronic depression" OR "treatment resistant depression" OR "persistent depression" OR dysthymi* OR depress*):ti,ab,kw	96586

Key: ti,ab,kw searches in title, abstract and author supplied keywords
