# Supplementary material for: Implementation of Evidence-Based Psychological Treatments to Address Depressive Disorders: A Systematic Review
Source: J Clin Med. 2025 Sep 8;14(17):6347. doi: 10.3390/jcm14176347 (PMC12429828; doi:10.3390/jcm14176347)
Supplement: Supplementary file 1 [file jcm-14-06347-s001.zip › Supplementary Materials_Table S1.pdf]

Table S1. Characteristics of the studies.

Authors	Year	Country	Design	Implementation outcomes	Patients	Implementers	Theoretical Framework	Intervention	Setting
**Asrat et al.,**	2021	South Africa	Pilot feasibility single-arm study	Acceptability, feasibility, and fidelity	People with HIV/AIDS / PLWHA and depression	Peer-counsellors	Not specified	Inter-Personal Therapy peer-administered	ART clinics
**Bina**	2017	Israel	Qualitative study	Appropriateness, acceptability, and feasibility	Women with postpartum depression	Social workers	Not specified	Inter-Personal Therapy	PC setting
**Bloomquist et al.,**	2017	USA	Hybrid design type II	Feasibility and fidelity	Adolescents with depression and/ or behaviour disorders and their parents	Lead practitioners and co-leaders	Not specified	Healthy Emotions Program	Community mental health setting
**Chen et al.,**	2019	USA	Hybrid design type II	Feasibility and fidelity	VA with depression	Psychologists and Masters level pre-doctoral psychology externs	Plan-Do- -Study-Act cycles'	Brief Dynamic Interpersonal Therapy	Veterans' Health Administration Medical Centre
**Clignet**	2016	Netherlands	Hybrid qualitative type III	Acceptability and fidelity	Old age patients with depression	Nurses	Not specified	Systematic Activation Method	Clinical units for old age psychiatry.
**Dear et al.,**	2020	Australia	Prospective longitudinal open	Acceptability	University students with depression and anxiety	Therapists =University Counselling and Psychological Services (CAPS) counsellors.	Not specified	UniWellbeing Course	University Counselling and Psychological Services
**Drozd et al.,**	2018	Norway	Hybrid design II -III	Acceptability, appropriateness, feasibility, and fidelity	Pregnant women with perinatal depression	Midwives and public health 122enurses	Active Implementation Framework	Mamma Mia	Primary care setting
**Eiraldi et al.,**	2019	USA	Hybrid design type II	Acceptability, feasibility, and fidelity	Students with depression	School staff and parents	Not specified	Group Cognitive Behavioural Therapy	Under resourced urban schools
**Fortney et al.,**	2012	USA	Nonrandomized small-scale multisite evaluation	Adoption, feasibility, fidelity, penetration, implementation cost, and sustainability	Veterans with depression	CBOCs staff members	RE-AIM and Plan- Do-Study- Act	Collaborative Care Management	Community Based Outpatient Clinics

**Fuchs et al.,**	2016	USA	Hybrid design type I	Acceptability, feasibility, penetration, and sustainability	Adults with depression and anxiety	Behavioural health assistants	Proctor's taxonomy	Mindfulness and acceptance-based group therapy	Primary care urban ambulatory family medicine practice
**Geraedts et al.,**	2014	Netherlands	Randomized Controlled Trial	Feasibility, fidelity, and penetration	Workers at companies with depression	Intervention providers = researchers	Not specified	Happy@Work	International companies
**Hadjistavropoulos et al.,**	2017	Canada	Process evaluation	Acceptability, adoption, appropriateness, feasibility, implementation cost and sustainability	Adults with depression	Therapists and managers	CFIR	Internet-delivered Cognitive Behaviour Therapy	Community mental health clinics
**Israel et al.,**	2013	Norway	Pilot Randomized Controlled Trial	Acceptability, feasibility, and fidelity	Adolescents with depression	Therapists	Not specified	Attachment Based Family Therapy	Hospital-based outpatient clinic
**Johnson et al.,**	2020	USA	Hybrid Design I	Acceptability and feasibility	Incarcerated population with depression	Counsellors	CFIR	Inter-Personal Therapy	State prison systems
**Kanine et al.,**	2021	UK	Hybrid Design II	Acceptability, feasibility, and fidelity	Adolescents with depression and anxiety	Therapists	Not specified	Inter-Personal Psychotherapy-Adolescent Skills Training	Urban Pediatric Care settings
**Karlin et al.,**	2019	USA	Hybrid Design I	Feasibility	Veterans with depression and co-occurring anxiety	Mental health providers	Not specified	Cognitive Behavioural Therapy	Kaiser Permanente health care system
**Kramer et al.,**	2008	USA	Randomized Controlled Trial	Acceptability, adoption, fidelity, and sustainability	Adolescents with depression	Therapists	Not specified	Cognitive Behavioural Therapy	Publicly-funded mental healthcare centres
**Lindholm et al.,**	2019	Finland	Longitudinal study	Acceptability, adoption, appropriateness, feasibility, penetration, and sustainability	Adults with depression and/or substance abuse	Psychologists and nurses	Normalization Process Theory and Proctors' Taxonomy	Behavioural Activation	Psychiatric units
**Lusk et al.,**	2011	USA	Preexperimental one-group pre- and posttest design	Feasibility and implementation cost	Adolescents with depression	Psychiatric nurses	Not specified	Creating Opportunities for Personal Empowerment	Community mental health clinic

**MacPherson et al.,**	2014	USA	Uncontrolled case study	Acceptability, adoption, appropriateness, feasibility, implementation cost, penetration, and sustainability	Children with depression and/or mood disorders	Therapists	Proctor's taxonomy	Multi-Family Psychoeducational Psychotherapy	Outpatient community clinics
**Mignona**	2014	USA	Hybrid design II	Acceptability, adoption, feasibility, and fidelity	Patients with depression and/or anxiety	Clinicians	PARISH	Brief Cognitive Behavioral Threapy	Primary Care of Veterans Affairs Hospitals
**Mignogna et al.,**	2018	USA	Hybrid design II	Fidelity	Patients with depression and/or anxiety	Multiprofessional group of providers	RE-AIM	Brief Cognitive Behavioral Therapy	Primary Care of Veterans Affairs hi
**Morrison et a.,**	2014	UK	Pilot implementation study	Acceptability and feasibility	Adults with depression	Psychological Wellbeing Practitioner	No specified	MindBalance	Improving Access to Psychological Therapies services
**Parhiala et al.,**	2019	Finland	Randomized Controlled Trial	Acceptability, feasibility, and fidelity	Adolescents with depression	Counsellors	No specified	Interpersonal Counselling and Brief Psychosocial support	Lower secondary schools
**Peterson et al.,**	2018	USA	Longitudinal study	Appropriateness and feasibility	Adults with depression and or/PTSD	Clinicians	No specified	Common Elements Treatment Approach	Public Behavioural Health
**Rasmussen et alk.,**	2019	Norway	Multi-site Randomized Controlled Trial	Feasibility, fidelity, and sustainability	Children with depression and /or anxiety	Health care and childcare professionals	EPIS	EMOTION	Schools
**Santucci et al.,**	2014	USA	Randomized Controlled Trial	Acceptability and feasibility	Students with depression and/or anxiety	Online, recruitment from behavioural medicine clinic	No specified	Beating the Blues	University-based health clinics
**Sit et al.,**	2022	China	Uncontrolled, single-arm pilot trial	Acceptability, adoption, feasibility, fidelity, and sustainability	Chinese young adults with depression and/or anxiety	Counsellors	RE-AIM	Step-by-Step	University
**Steinfeld et al.,**	2009	USA	Implementation study	Adoption, feasibility, and sustainability	Patients with depression and anxiety	Mental health providers	No specified	Cognitive–Behavioural Therapy	Group Health Cooperative

**Walser et al.,**	2013	USA	Large scale, multi-site, multi-cohort dissemination	Feasibility	Veterans with depression and anxiety	Mental health clinicians	No specified	Acceptance and Commitment Therapy for Depression	Veterans' Health Administratio n
**Wilfley et al.,**	2020	USA	A Cluster-Randomized Trial	Fidelity	University students with depression and eating disorders.	Therapists	No specified	Inter-Personal Psychotherapy	College Counselling Centre

CFIR= Consolidated Framework for Implementation Research; EPIS: Exploration, Preparation, Implementation and Sustainment; HIV/AIDS/PLWHA= Human Immunodeficiency Virus/ Acquired Immunodeficiency Syndrome/ People living with HIV/AIDS; PARISH= Promotion, Action on Research Implementation; PC= Primary Care; PTSD= Post-Traumatic Stress Disorder; RE-AIM= Reach, Effectiveness, Adoption, Implementation and Maintenance; US= United States; VA=Veterans Affairs.
