# Supplementary material for: Implementation of Evidence-Based Psychological Treatments to Address Depressive Disorders: A Systematic Review
Source: J Clin Med. 2025 Sep 8;14(17):6347. doi: 10.3390/jcm14176347 (PMC12429828; doi:10.3390/jcm14176347)
Supplement: Supplementary file 1 [file jcm-14-06347-s001.zip › Supplementary Materials_Table S2.pdf]

Table S2. Assessment according to the CFIR framework for study implementation.

CFIR	STUDIES																													
**Domains and constructs of the CFIR**	**56**	**64**	**7**	**5**	**65**	**53**	**72**	**54**	**67**	**62**	**58**	**77**	**47**	**94**	**60**	**48**	**76**	**55**	**49**	**63**	**66**	**68**	**71**	**50**	**61**	**69**	**51**	**52**	**73**	**74**	**75**
**I. INTERVENTION CHARACTERISTICS**																															
**A. Intervention source**												X																			
**B. Evidence Strength & Quality**	X		X	X					X			X	X	X			X	X	X	X						X			X	X	X
**C. Relative advantage**				X								X	X	X			X	X	X												
**D. Adaptability**	X	X	X	X	X		X	X	X			X				X		X	X	X	X	X		X	X	X			X		
**E. Trialability**				X		X	X	X	X		X	X	X	X	X		X				X	X	X	X	X		X	X			X
**F. Complexity**				X	X			X				X	X				X	X	X		X			X		X					
**G. Design Quality & Packaging**	X					X						X								X	X				X						
**H. Cost**									X			X		X					X	X									X		
**TOTAL:**	**3**	**1**	**2**	**5**	**2**	**2**	**2**	**3**	**4**	**0**	**1**	**8**	**4**	**4**	**1**	**1**	**4**	**4**	**5**	**4**	**4**	**2**	**1**	**3**	**3**	**3**	**1**	**1**	**3**	**1**	**2**

**II. OUTER SETTING**																															
**A. Patient Needs & Resources**	X	X		X		X	X	X	X			X		X	X				X		X				X	X		X			
**B. Cosmopolitanism**							X	X	X	X		X																			
**C. Peer Pressure**																															
**D. External Policy & Incentives**				X										X																	
**TOTAL:**	**1**	**1**	**0**	**2**	**0**	**1**	**2**	**2**	**2**	**1**	**0**	**2**	**0**	**2**	**1**	**0**	**0**	**0**	**1**	**0**	**1**	**0**	**0**	**0**	**1**	**1**	**0**	**1**	**0**	**0**	**0**
**III. INNER CONTEXT**																															
**A. Structural Characteristics**									X			X					X							X			X	X	X		
**B. Networks & Communications**												X	X	X	X							X				X					
**C. Culture**				X				X				X		X						X					X	X			X		
**D. Implementation Climate**	X	X		X			X	X	X			X	X	X			X			X	X	X							X		
**1. Tension for Change**	X											X		X												X			X		
**2. Compatibility**	X	X			X			X	X			X		X				X	X	X					X	X	X		X		

**3. Relative Priority**											X		X					X													
**4. Organizational Incentives & Rewards**							X		X	X		X					X			X				X					X		
**5. Goals and Feedback**			X					X	X			X		X		X				X						X					
**6. Learning Climate**			X		X		X				X	X		X		X	X				X	X			X	X			X	X	
**E. Readiness for Implementation**	X								X			X	X	X												X					
**1. Leadership Engagement**	X		X									X	X			X	X									X					
**2. Available Resources**	X	X		X	X		X	X	X	X	X	X	X	X	X	X	X	X	X	X	X	X		X	X	X		X	X	X	X
**3. Access to knowledge and information**	X		X	X	X	X	X	X	X		X	X	X	X		X		X		X	X	X			X	X		X	X	X	X
**TOTAL:**	**7**	**3**	**4**	**4**	**4**	**1**	**5**	**6**	**8**	**2**	**3**	**1**	**6**	**1**	**2**	**5**	**6**	**3**	**3**	**7**	**4**	**5**	**0**	**3**	**5**	**1**	**2**	**3**	**9**	**3**	**2**
**IV. CHARACTERISTIC OF INDIVIDUALS**																															
**A. Knowledge & Beliefs about the Intervention**	X	X		X	X		X					X	X	X	X	X	X		X			X			X				X	X	X

**B. Self-efficacy**			X		X			X				X				X	X				X	X		X	X	X			X	X	X
**C. Individual Stage of Change**			X				X					X	X			X														X	
**D. Individual Identification with Organization**												X		X												X					
**E. Other Personal Attributes**	X		X	X				X				X	X	X										X		X					X
**TOTAL:**	**2**	**2**	**3**	**2**	**2**	**0**	**2**	**2**	**0**	**0**	**0**	**5**	**3**	**3**	**1**	**3**	**2**	**0**	**1**	**0**	**1**	**3**	**0**	**2**	**2**	**3**	**0**	**0**	**2**	**3**	**3**
**V. PROCESS**																															
**A. Planning**				X			X	X	X			X	X			X		X		X	X						X		X		
**B. Engaging**	X	X	X	X		X	X	X	X		X	X				X		X			X	X		X	X	X			X	X	X
1. Opinion Leaders																															
**2. Formally appointed internal implementation leaders**	X		X			X	X	X	X	X		X	X		X		X	X			X	X		X	X	X	X		X	X	X
**3. Champions**					X				X								X			X	X	X						X	X		

**4. External Change Agents**			X	X	X			X				X	X		X	X					X	X								X	
**C. Executing**	X		X		X			X	X			X			X	X		X			X			X		X					
**D. Reflecting & Evaluating**	X		X	X	X		X	X	X			X	X		X	X	X	X		X	X	X		X	X	X		X	X	X	X
**TOTAL:**	**4**	**1**	**5**	**4**	**4**	**2**	**4**	**6**	**6**	**1**	**1**	**6**	**4**	**0**	**4**	**5**	**3**	**5**	**0**	**3**	**7**	**5**	**0**	**4**	**3**	**4**	**2**	**2**	**5**	**4**	**3**
